# Stingray Envenomation: Consequences of an Embedded Spine

**DOI:** 10.7759/cureus.38885

**Published:** 2023-05-11

**Authors:** Pablo Mora-Zamacona, Ruth N Águila-Ramírez, Mauricio Muñoz-Ochoa, Xchel A Pérez-Palafox, Yanet Sepúlveda De La Rosa, Christine J Band-Schmidt, Víctor H Cruz-Escalona

**Affiliations:** 1 Bioeconomía Pesquera y Acuícola, Centro de Investigaciones Biológicas del Noroeste, La Paz, MEX; 2 Desarrollo de Tecnologías, Instituto Politécnico Nacional, Centro Interdisciplinario de Ciencias Marinas, La Paz, MEX; 3 Pesquerías y Biología Marina, Instituto Politécnico Nacional, Centro Interdisciplinario de Ciencias Marinas, La Paz, MEX; 4 Evolution, Behaviour, and Environment, School of Life Sciences, University of Sussex, Brighton, GBR; 5 Plancton y Ecología Marina, Instituto Politécnico Nacional, Centro Interdisciplinario de Ciencias Marinas, La Paz, MEX

**Keywords:** stingray, barb, flap, graft, necrosis, venom

## Abstract

Rays and skates are fish with flattened, pancake-shaped bodies that frequent shallow water, where they often lie hidden under the sand. Some of the batoid species are characterized by a stinger with serrated edges, which is covered by a tegument made up of specialized cells that secrete toxins and enzymes with proteolytic activity. Stingray injuries to humans are common in warm coastal regions. In this report, we present a case of an injury due to the insertion of a barb from a Pacific cownose ray, Rhinoptera steindachneri*.* We assess the tissue complications due to the retention of the spine in the foot, the subsequent infection that caused tissue necrosis, and the reconstructive surgery performed. Based on previous experience, we highly recommend performing diagnostic procedures such as soft tissue radiographs and MRI to ensure the absence of the barb within the wound and thereby avoid further complications. Current textbook treatment is based on limited scientific studies, case reports, and successful clinical treatment of many victims.

## Introduction

Stingrays are a subset of the cartilaginous fish commonly known as rays; these are members of the class Chondrichthyes, which also includes sharks and skates. Rays and skates (batoids) are flattened cartilaginous fish with muscular wings that share their ancestral roots with sharks and can range in size from several inches to 12 feet in length [1]. They are widely distributed in the temperate and tropical regions of the world [1] and most of these species inhabit the oceans, except for the Potamotrygonidae, which is restricted to freshwater habitats [1]. Some of the batoid species are characterized by a spiny apparatus (barb) with serrated edges used as a defense mechanism against predators [2]. The barb is covered by a tegument made up of specialized cells that secrete toxins and enzymes with a proteolytic activity that promotes inflammatory processes and necrosis of the affected tissue [3,4].

Batoids are responsible for a significant number of injuries to humans, with around 2000 cases reported annually in the United States of America [5-6]. Most injuries caused by rays and skates occur on beaches when people accidentally step on them or when they are captured and handled by fishermen [3,7-9]. A stingray injury has two components: (1) a traumatic injury consisting of a penetrating stab wound, and (2) envenomation from toxin released into the wound [2]. The traumatic component can be a puncture wound or laceration and is similar to an injury caused by a serrated, stiletto-type knife entering the body. Due to the barb's tendency to fragment upon entry into the body, the remains left inside the wound can lead to further complications. Envenomation occurs when the integumentary sheath surrounding the barb ruptures on penetration. Pieces of the toxin-containing sheath are usually deposited in the subcutaneous tissue [2].

The predominant local symptom of stingray injury is an immediate, severe stabbing pain, which may intensify for up to two hours after the sting and radiate to the entire limb [7,9]. Some studies have shown that stingray venom activity induces edema formation that can persist for 48 hours after the sting, and while the severity of the injury is variable, it is related to the amount of venom inoculated [3,10]. However, superficial lacerations are the most common type and the wound can bleed with localized edema formation. General symptoms are inconsistent and may involve lipothymia (fainting), digestive disorders, and arterial hypotension. If the wound becomes infected, it may cause hemolysis and, in severe cases, lead to shock and cardiorespiratory arrest [3,11]. Extensive tissue necrosis is rarely observed, but when it happens, it requires reconstruction procedures [12].

## Case presentation

**Figure 1:**
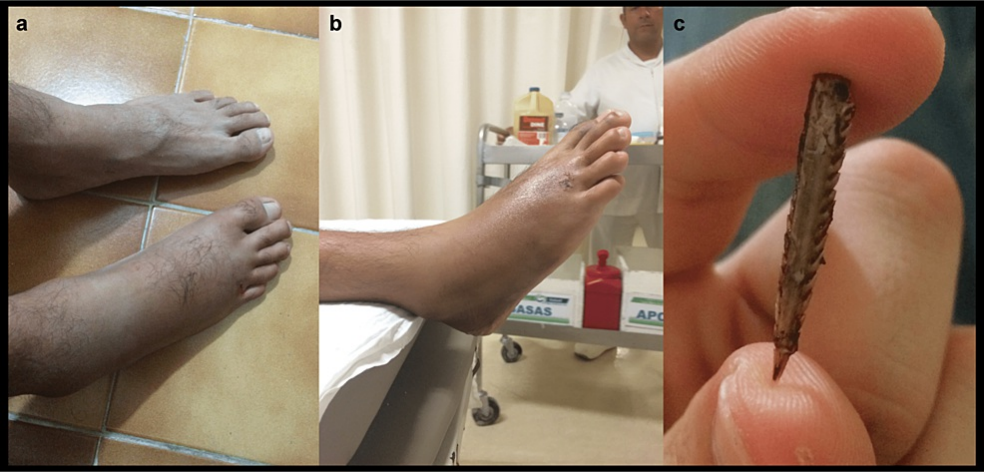
(a) State of the affected foot five days after the sting, (b) inflammatory process caused by the wound, and (c) barb extracted from the affected foot

The patient was a 25-year-old man with a barb from a Pacific cownose ray, Rhinoptera steindachneri, embedded in the instep of his right foot, at the base of the fourth toe at the distal portion of the fourth metatarsal. After the accident, the specimen had been checked and apparently had a complete barb. The foot had been only subjected to an external visual inspection, with analgesics recommended in case of pain, but no treatment had been administered. The foot had become swollen after one day, and the swelling progressively increased over time (Figure 1a). Additionally, the swelling was not confined to the affected area but also extended to the ankle (Figure 1b).

**Figure 2:**
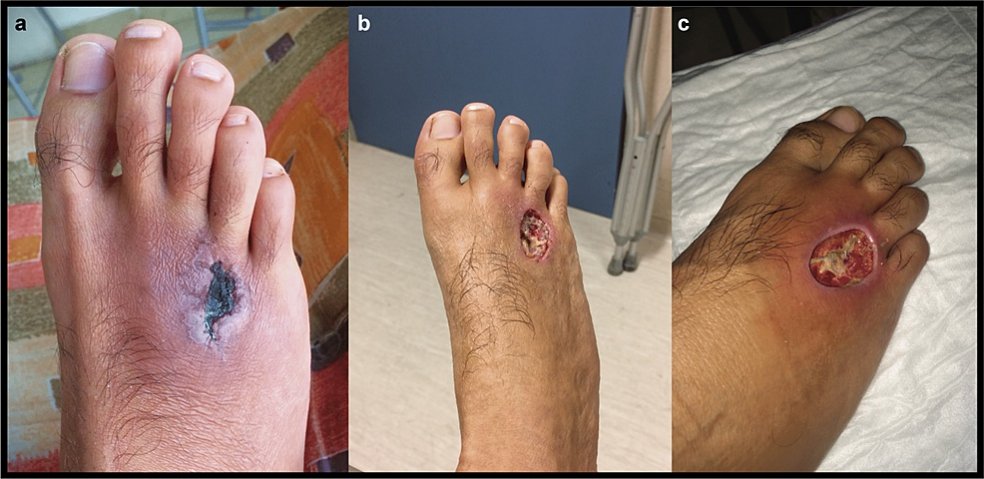
(a) Appearance of the foot two weeks after the extraction of the barb. Darkened skin appeared around the affected area. (b-c) Foot after surgery, with the necrotic tissue removed

A plain radiograph/X-ray of the right foot was taken, revealing that the barb of the stingray was still lodged inside the foot. Surgery was performed to remove the barb (Figure 1c). Oral antibiotics, anti-inflammatory medications, and analgesics were administered. After the surgery, the inflammation began to progressively decrease; however, a change in skin coloration to a darker tone was observed around the affected area (Figure 2a). Following this, surgical intervention was performed to remove the infected and necrotic tissue in the affected area; the amount of tissue removed during the intervention was substantial (Figure 2b).

**Figure 3:**
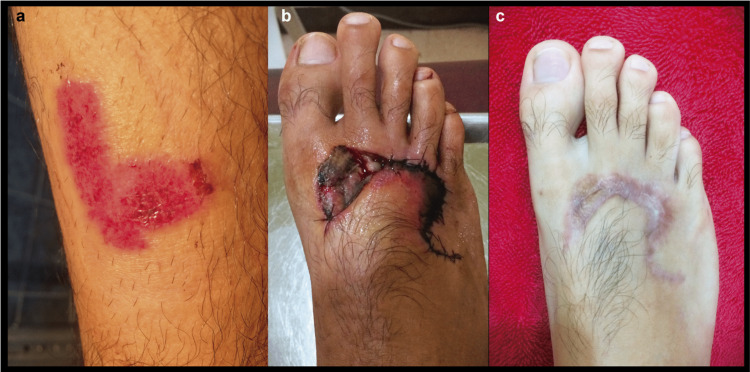
(a) Right calf from where healthy tissue was removed for the graft procedure, (b) appearance of the foot after the flap and graft surgical procedures, and (c) appearance of the scarred foot two months from surgery

Since the wound was taking longer than expected to heal, the opinion of a physician specializing in cosmetic surgery was sought. The specialist identified the presence of necrotic tissue in the affected area and a brief surgical intervention was performed to remove the infected tissue. Due to the removal of the tissue, the tendons of the third and fourth toes were exposed (Figure 2c). Daily cleaning of the wound and application of ointments to facilitate granulation were carried out for about a month. A final surgical intervention was performed, which consisted of two processes. The first one comprised a flap surgery procedure that partially separated the skin and healthy tissues to place them over the wound. The second procedure was a skin graft that involved removing a portion of healthy skin from the right calf (Figure 3a) and placing it over the wound inflicted by the flap surgery (over the first and second metatarsals; Figure 3b). After a couple of months, the healing of the foot was complete, and its mobility was maintained. The only remnants of the wound were increased skin sensitivity and the presence of a scar (Figure 3c).

## Discussion

Stingrays are organisms that rarely exhibit aggressive behaviors. However, when they feel threatened, they defend themselves using a barb located on the whip-shaped tail. This movement is a defensive reflex and is characterized by throwing the tail backward or sideways to reach the potential enemy’s body [3]. In marine environments, most of the lesions are produced by small and round stingrays of the family Urolophidae, which are the most abundant type. However, in our case, the injury was caused by an active midwater species, the Pacific cownose ray (Rhinoptera steindachneri). This species belongs to the order Myliobatiformes and is the only representative of the Rhinopteridae family, found in the Eastern Pacific. It inhabits shallow waters, especially over soft bottoms, and performs seasonal migrations related to water temperature. The barb’s characteristics are as follows: total spine lengths in males: 45 mm standard total length (STL), in females: 21 mm STL; 57 total serrations in males, 41 in females; pb/STL averages are 69% in both sexes; 1- to 3-mm space on both sides of the base [13].

The described incident occurred during a field sampling of stingrays in Bahía de La Paz. During the sampling, a stingray specimen injured the patient as a defensive reflex action, leading to the described injury. Local fishermen have commented that they have developed immunity toward stingrays’ venom over the years. When injuries occur, the traditional treatment is to spread hot motorboat oil or common insecticides over the injury. According to them, this eliminates or reduces severe complications. The effect of these substances that may inhibit the effect of the venom of the stingray or how fishermen develop a natural immunity remains to be understood; the possible negative effects of coming into contact with these substances also need to be studied.

The venomous barb of these fish has not been extensively studied, unlike other freshwater species. This is due to several reasons, such as the absence of venom glands that can be milked (as is done with snakes) and the fact that the venom produced by rays is usually unstable, heat-labile, and water-soluble. Russell et al. [4] demonstrated that these venoms are composed of several cardiotoxic enzymatic proteins with arrhythmogenic and cardiac depressant effects. In species such as Dasyatis sephen, Dolichocephala guttata, and Aetobatis narinari, their venom has been found to contain various components, including serotonin, metalloproteinases, 5’nucleotidase, and phosphodiesterases. These substances have vasomotor (vasoconstriction), proteolytic, gelatinolytic, and hyaluronidase activity [5,14,15,16,17], which produces a large inflammatory reaction, attracting lymphocytes and eosinophils, potentially leading to tissue necrosis [8].

The local effects will depend on the type of lesion caused, from a superficial laceration to inflammation by direct lesion of the venom or retention of the barb in the soft tissues [3,5,11,17]. In the latter case, the barb of these animals is formed by a cartilaginous structure known as vasodentin and is covered by an epithelium that has many specialized cells that produce venom [4]. When the barb or fragments of it are retained in the victim’s tissues, it can produce an intense inflammatory process causing some major complications such as lymphangitis, tissue necrosis of the peripheral area and involvement of all neighboring tissues, septicemia, osteomyelitis, or granulomatous foreign body reaction [3,5,11,17,18].

A procedure that should be considered following a sting is the performance of radiographs of the affected areas [8,19] since there could be remnants of the barb, and, as in the case of our patient, many further complications could be avoided. However, when a barb is not apparent, MRI may offer a more appropriate choice. MRI may be indicated when more areas are affected and for further visualization of suspected hypodense material, though at a higher cost [14,19]. Biopsies of stingray injuries are rarely performed, and the findings are not well characterized [9,12,17]. One case biopsied two months after the injury showed a large zone of pluricellular necrosis with superficial ulceration and granulomatous inflammation; the barb venom was most likely responsible for the pattern of necrosis noted in the biopsy [20].

As mentioned before, the type of lesion caused by the barb and the amount of venom inoculated may determine the severity of the symptoms. Moreover, as presented in the clinical case, the retention of the barb or fragments could lead to additional complications. Skin damage could be caused by an immune response (inflammatory process) to either the residual tissue or some compound of a protein nature, or by bacterial infection of an unknown nature. In many cases, external inspection of the lesion may not be enough [20]; thus, we highly recommend the performance of plain radiograph/X-ray, MRI, or alternative diagnostic procedures to ensure the absence of the barb within the tissue to avoid further complications.

## Conclusions

Stingray barbs generally cause minor injuries with localized symptoms. However, in some cases, such as the one presented, the injury sustained can be more serious and cause significant damage if not treated properly. In such cases, particularly when early osculation of the wound and removal of the stinger or bone fragments are not performed, more aggressive surgical debridement is required. In our case, the timely intervention of the surgeons and the surgical procedures performed managed to prevent the patient from having more serious complications that could have led to the amputation of the foot.

## References

[1] (2016). Rays of the World. https://ebooks.publish.csiro.au/content/rays-world.

[2] Meyer PK (1997). Stingray injuries. Wilderness Environ Med.

[3] Diaz JH (2008). The evaluation, management, and prevention of stingray injuries in travelers. J Travel Med.

[4] Russell FE (1953). Stingray injuries: a review and discussion of their treatment. Am J Med Sci.

[5] Germain M, Smith KJ, Skelton H (2000). The cutaneous cellular infiltrate to stingray envenomization contains increased TIA+ cells. Br J Dermatol.

[6] Falk DP, Metikala S, Lopez VS, Stein M, Mahmoud K, Chao W (2019). Late presentation of a retained stingray spine in the plantar medial hindfoot. Foot Ankle Orthop.

[7] Grainger CR (1980). Occupational injuries due to sting-rays. Trans R Soc Trop Med Hyg.

[8] Fenner PJ, Williamson JA, Skinner RA (1989). Fatal and non-fatal stingray envenomation. Med J Aust.

[9] Auerbach PS (1995). Wilderness Medicine: Management of Wilderness and Environmental Emergencies.

[10] Kimura LF, Prezotto-Neto JP, Antoniazzi MM, Jared SG, Santoro ML, Barbaro KC (2014). Characterization of inflammatory response induced by Potamotrygon motoro stingray venom in mice. Exp Biol Med (Maywood).

[11] Clark AT, Clark RF, Cantrell FL (2017). A retrospective review of the presentation and treatment of stingray stings reported to a poison control system. Am J Ther.

[12] (2023). Stingray sting. https://www.statpearls.com/point-of-care/29504.

[13] Schwartz FJ (2008). A survey of tail spine characteristics of stingrays frequenting African, Arabian to Chagos-Maldive Archipelago waters. J North Carolina Acad Sci.

[14] Cazorla D, Loyo J, Lugo L, Acosta M (2009). Clinical, epidemiological and treatment aspects of 10 cases of saltwater stingray envenomation (Article in Spanish). Rev Invest Clin.

[15] Rajeshkumar RK, Vennila R, Karthikeyan S, Prasad NR, Arumugam M, Velpandian T, Balasubramaniam T (2015). Antiproliferative activity of marine stingray Dasyatis sephen venom on human cervical carcinoma cell line. J Venom Anim Toxins Incl Trop Dis.

[16] Ziegman R, Alewood P (2015). Bioactive components in fish venoms. Toxins (Basel).

[17] Fuochi V, Li Volti G, Camiolo G (2017). Antimicrobial and anti-proliferative effects of skin mucus derived from Dasyatis pastinaca (Linnaeus, 1758). Mar Drugs.

[18] Mix FM, Sucher BM, Brooks JE (2021). Stingray-induced tarsal tunnel syndrome. Cureus.

[19] Barber GR, Swygert JS (2000). Necrotizing fasciitis due to Photobacterium damsela in a man lashed by a stingray. N Engl J Med.

[20] O'Malley GF, O'Malley RN, Pham O, Randolph F (2015). Retained Stingray Barb and the Importance of Imaging. Wilderness Environ Med.

